# Exploring the Research Landscape of Transcranial Direct Current Stimulation in Stroke: A Bibliometric Review

**DOI:** 10.7759/cureus.76510

**Published:** 2024-12-28

**Authors:** Ayesha Juhi, Rintu Kumar Gayen, Manul Das, Chanchal Goyal, Shreya Sharma, Md Asif Khan, Pritam Chaudhary, Himel Mondal

**Affiliations:** 1 Physiology, All India Institute of Medical Sciences, Deoghar, Deoghar, IND; 2 Electronics and Communication Engineering, Institute of Engineering and Management, Kolkata, IND; 3 Clinical Research, Central Institute of Psychiatry, Ranchi, Ranchi, IND; 4 Centre for Evidence for Guidelines, Indian Council of Medical Research, New Delhi, IND; 5 Neuromodulation Laboratory, All India Institute of Medical Sciences, Deoghar, Deoghar, IND; 6 Descriptive Research Division, Indian Council of Medical Research, New Delhi, IND

**Keywords:** bibliometric analysis, citation analysis, cognitive recovery, motor recovery, neuroplasticity, research trends, stroke rehabilitation, stroke therapy, tdcs, transcranial direct current stimulation

## Abstract

Transcranial direct current stimulation (tDCS) has gained significant attention as a potential therapeutic tool in stroke rehabilitation, promoting neuroplasticity and enhancing motor and cognitive recovery. Despite growing research, the field's evolution and key trends remain underexplored. This study aims to perform a bibliographic analysis of publications related to tDCS and stroke rehabilitation to assess the growth of the field. Published literature was searched in PubMed and Web of Science (WOS) on 10 December 2024. We used the keywords “transcranial direct current stimulation” and “stroke” to collect studies without any time limitation. Articles found in WOS were used to get trends, and a search from PubMed was used to analyze co-occurrences in VOSviewer, version 1.6.20 (Centre for Science and Technology Studies, Leiden University, the Netherlands). A total of 1,598 articles were found in WOS, and 1,300 were found in PubMed. As there was overlapping of subject categories, countries, and affiliations, the total number differed in calculating the percentages. The analysis revealed significant growth in publications on tDCS and stroke rehabilitation, peaking at 137 publications in 2022. Most studies focus on neuroscience (860, or 30.6%), clinical neurology (562, or 20%), and rehabilitation (244, or 8.68%). The United States leads contributions (519, or 22.4%), followed by Germany (249, or 10.7%) and China (179, or 7.7%). Publications are concentrated among major publishers like Elsevier (358, or 22.4%) and key journals such as *Brain Stimulation* and *Frontiers in Human Neuroscience*. English dominates as the primary language (1,572, or 98.37%). Research emphasizes tDCS’s role in motor recovery and brain plasticity in stroke rehabilitation. This bibliometric analysis highlights a substantial and growing interest in tDCS for stroke rehabilitation, with a steady increase in publications. The focus of research predominantly lies in neuroscience, clinical neurology, and rehabilitation, reflecting the central role of tDCS in advancing stroke recovery and brain plasticity. The concentration of publications among major publishers and journals underscores the prominence of specific platforms in disseminating tDCS research. More research from developing countries is needed to achieve a balanced geographical diversity on this topic.

## Introduction and background

Stroke is a leading cause of death (with an annual mortality rate of about 5.5 million) and long-term disability worldwide, with significant social and economic burdens on patients, caregivers, and healthcare systems [1]. Despite advancements in acute stroke management, such as thrombolysis and thrombectomy, a large proportion of survivors experience residual motor, cognitive, and functional impairments [2]. Rehabilitation plays a critical role in stroke recovery by promoting neuroplasticity [3]. However, traditional rehabilitation methods often yield variable outcomes, and the need for innovative adjunctive therapies to enhance recovery remains paramount [4].

Transcranial direct current stimulation (tDCS), a non-invasive neuromodulation technique, has emerged as a promising tool in stroke rehabilitation [5]. By delivering a low-intensity direct current to the scalp, tDCS modulates cortical excitability and promotes plasticity in targeted brain regions. Studies suggest that tDCS can facilitate motor learning, enhance recovery of motor function, and improve cognitive processes in stroke patients [6]. The versatility of tDCS, including its safety profile, cost-effectiveness, and potential for home-based applications, has garnered significant interest among clinicians and researchers [7].

Despite the growing body of literature on tDCS and stroke, challenges remain in understanding its optimal protocols, mechanisms of action, and long-term efficacy [8]. Over the years, a growing body of research has explored the applications, mechanisms, and outcomes of tDCS in stroke rehabilitation. A bibliographic analysis of research publications on tDCS and stroke can provide valuable insights into the evolution of this field, identifying influential studies, key contributors, and emerging trends.

With this background, this study aims to conduct a comprehensive bibliographic analysis of research publications on tDCS in the context of stroke rehabilitation. The scope includes examining the temporal trends in publications, identifying influential authors, institutions, and journals, and mapping global research collaborations. This analysis will serve as a valuable resource for researchers and clinicians who are researching or planning to conduct studies on the use of tDCS for stroke recovery.

## Review

Materials and methods

Study Design

This study performed a systematic bibliometric analysis of research publications focusing on tDCS in stroke rehabilitation. This cross-sectional study was conducted in December 2024 with publicly available data and subscription-based data from two bibliographic databases: Web of Science (WOS) and PubMed. Specific objectives included assessing publication trends over time and identifying key subject categories, countries, publishers, journals, affiliations, and co-occurrences.

Search Strategy

A search was conducted across two bibliographic databases - PubMed and WOS. We could only include these two databases due to the feasibility of using the data for analysis and logistical limitations. We did not have access to databases like Embase or Scopus. We used the keywords “transcranial direct current stimulation” and “stroke.” Specifically, in WOS, the keywords were searched as “(ALL = (Transcranial Direct Current Stimulation)) AND ALL = (Stroke).” In PubMed, it was searched as “Transcranial Direct Current Stimulation” AND “Stroke.” We included all types of articles published to date to get the overall publication trends.

Data Collection

**Figure 1:**
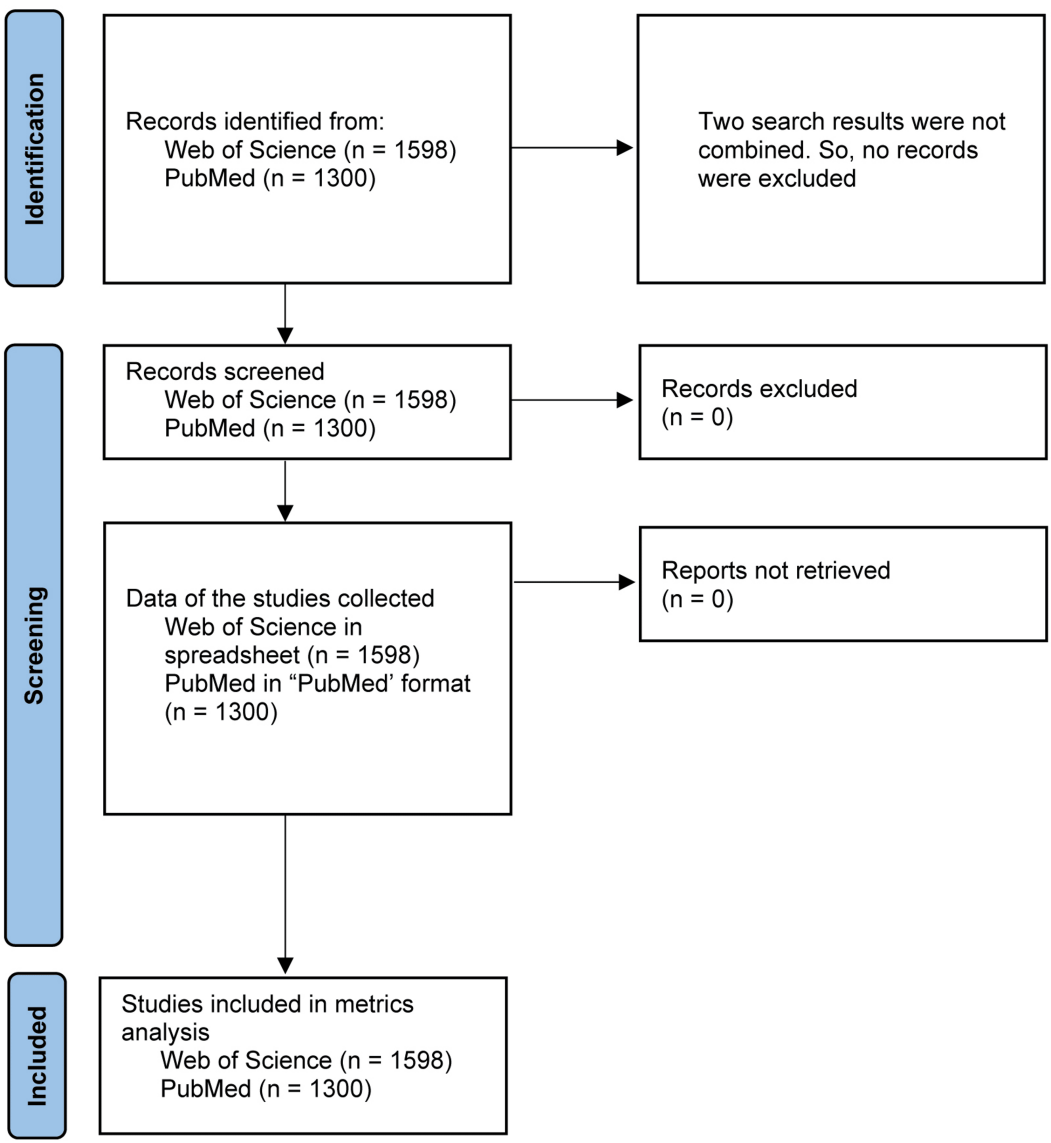
Flow chart showing the number of articles used in this analysis (similar to Preferred Reporting Items for Systematic Reviews and Meta-Analyses) n: number

We collected the data on year-wise publication, category or broad subject in which studies were published, countries from which authors published the articles, publishers, journals, language, affiliations, and co-occurrence of key terminologies in the published literature. We downloaded the data in a suitable format (spreadsheet from WOS and PubMed format from PubMed). The data were collected on 10 December 2024. We found a total of 1,598 articles in WOS and 1,300 in PubMed. We screened the data to see if the articles were related to tDCS and stroke, and all were found to be included in the bibliometric analysis. We did not combine the results of the two databases, as we used the two databases for two different reasons (WOS for all analysis except co-occurrence of keywords analysis in VOSviewer). We followed the guidelines of the Preferred Reporting Items for Systematic Reviews and Meta-Analyses (PRISMA) for data handling wherever possible, and the flow chart is shown in Figure 1.

Data Analysis

Data were presented in number, percentage, mean, median, and first quartile (Q1) and third quartile (Q3). Although the studies found from WOS were 1,598, in many cases, a different number was yielded. For example, a study can be categorized into different subject categories. Hence, although the studies were 1,598, the sum of the category-wise number of publications was 2,810. During the data presentation in tables and figures, we have mentioned the number with which the percentages were calculated. To present the results clearly, graphical representations were created using Microsoft Excel 2010 (Microsoft® Corp., Redmond, WA, USA) and VOSviewer version 1.6.20 (Centre for Science and Technology Studies, Leiden University, the Netherlands).

Ethical Considerations

All data used in this study were derived from publicly available sources. Ethical guidelines for bibliographic analysis were strictly followed to ensure transparency and reproducibility. As there were no study participants in the study, no raw data were presented, and only the analyzed output was presented. Therefore, this study does not require any ethical clearance.

Results

**Figure 2:**
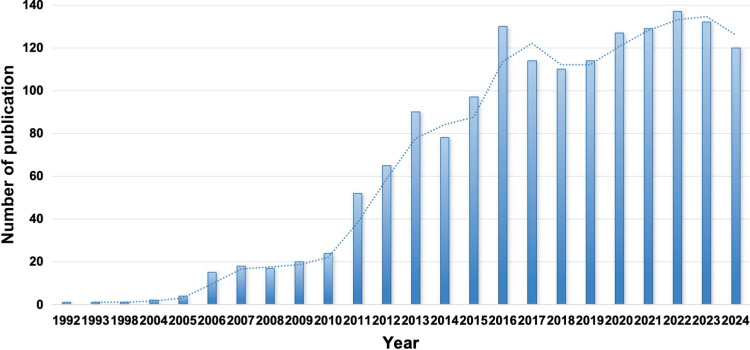
Year-wise publication on transcranial direct current stimulation in stroke

The year-wise publication trends demonstrate a marked increase in research interest over the decades. A steady upward trajectory followed, with a spurt between 2011 (52 publications) and 2016 (130 publications), indicating expanding scientific interest and broader adoption of tDCS in stroke research. From 2016 onward, the annual publication rate stabilized at a high level, peaking in 2022 with 137 articles. The trend over the years is shown in Figure 2.

**Figure 3:**
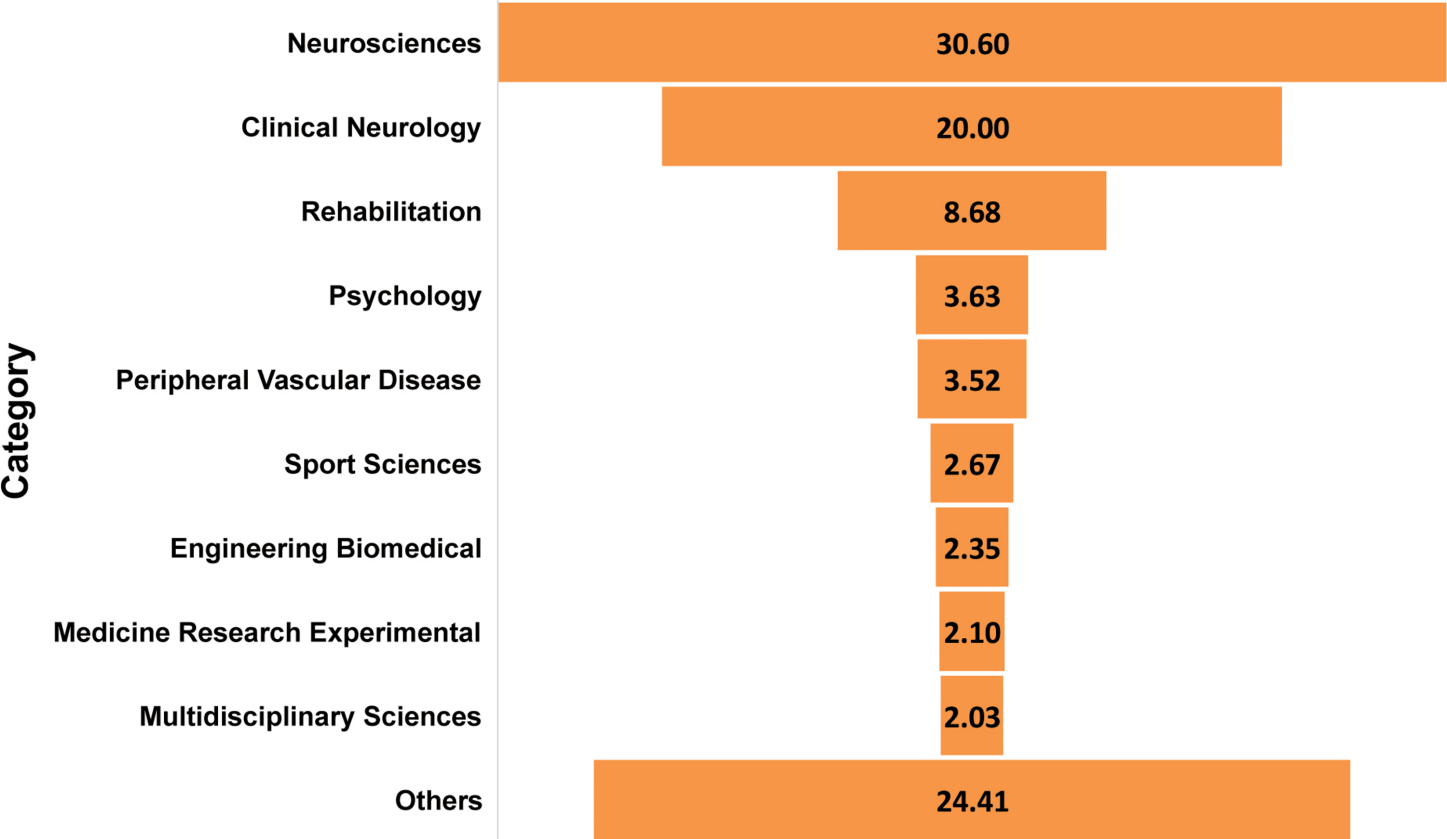
Studies according to Web of Science categories The bar chart shows the percentage of studies, calculated based on the total number of 2,810. Categories with less than 2% were grouped into the “others” category.

When the studies were categorized according to subjects, they were divided into a total of 83 categories. The top categories are shown in Figure 3.

The majority of the studies were in the neuroscience category, with 860 (30.6%), followed by clinical neurology with 562 (20%). Rehabilitation was the next category, with 244 (8.68%) studies. Contributions less than 2% were in the “others” group, which included categories such as general internal medicine, psychiatry, behavioral science, neuroimaging, and physiology, among others.

**Figure 4:**
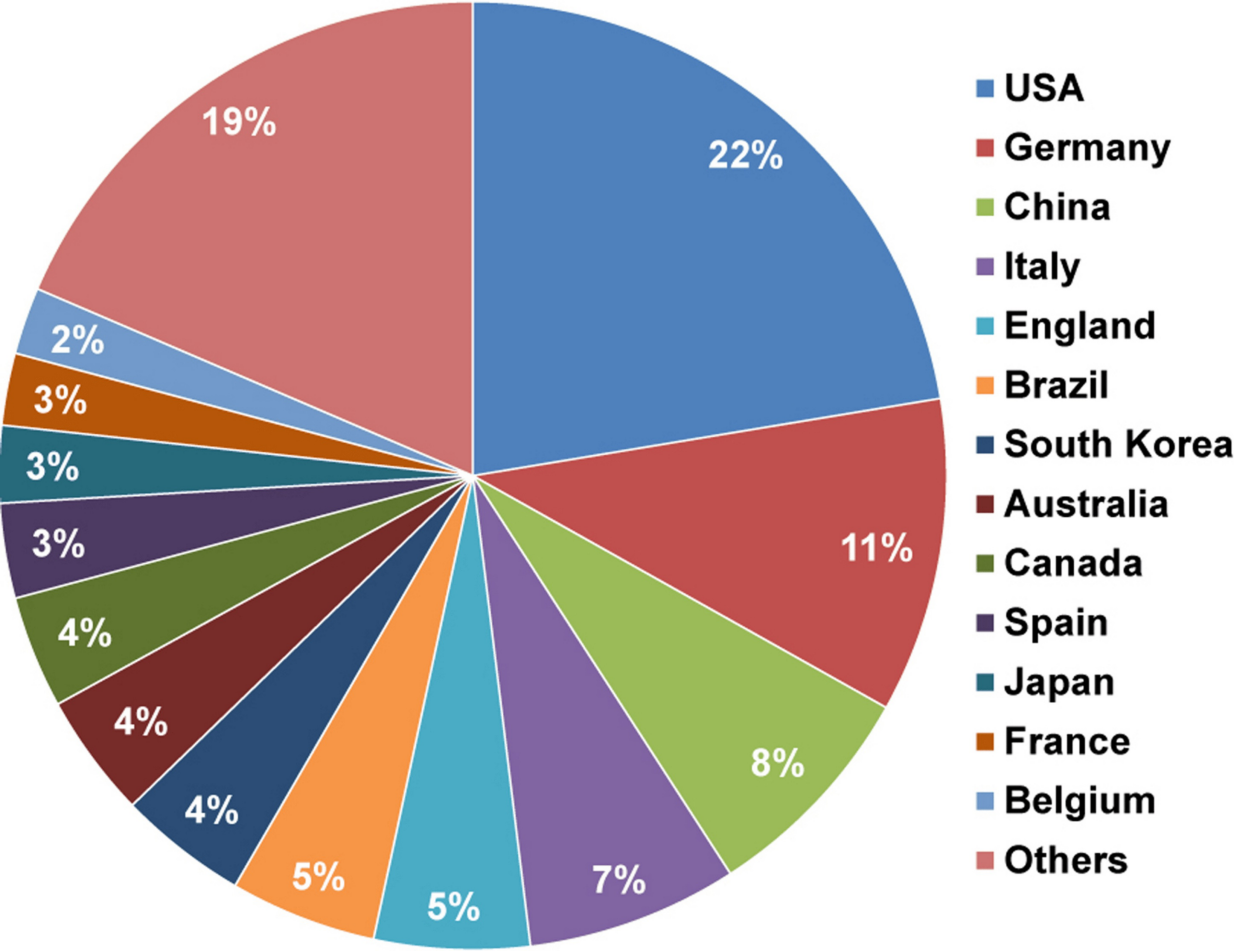
Country-wise distribution of the publications The pie chart shows the percentage of studies, calculated based on the total number of 2,318. Categories with less than 2% were grouped into the “others” category.

A total of 69 countries have contributed literature on tDCS in stroke. The country-wise data reveal that the United States leads significantly, contributing 519 (22.4%) of the total studies. Germany follows with 249 (10.7%) studies, and China contributes 179 (7.7%), while Italy contributes 167 (7.2%) studies. Any country whose contribution is below 2% is shown in the “others” category. The distribution is shown in Figure 4.

A total of 95 publishers contributed to the literature, with a median of 2 (Q1-Q3: 1-6) publications per publishing house. The top 10 publishers are shown in Table 1.

**Table 1:** Top 10 publishers publishing papers related to transcranial direct current stimulation in stroke The percentage was calculated based on the total number of 1,598. SA: Société Anonyme, MDPI: Multidisciplinary Digital Publishing Institute, IOS: International Scientific, Technical, and Medical publishing house

Publisher	Number (%)
Elsevier	358 (22.4)
Frontiers Media SA	178 (11.14)
Springer Nature	168 (10.51)
Lippincott Williams & Wilkins	133 (8.32)
Wiley	115 (7.2)
Taylor & Francis	84 (5.26)
Sage	78 (4.88)
MDPI	75 (4.69)
IOS Press	71 (4.44)
Nature Portfolio	25 (1.56)

These 10 publishers host 1,285 (80.41%) of the total publications. Elsevier dominates the field, accounting for 358 (22.4%) of the studies, followed by Frontiers Media SA, with 178 (11.14%) and Springer Nature, with 168 (10.51%) articles. This distribution highlights the concentration of research output in a few major publishing houses. The other 86 publishers collectively contribute 273 (19.59%) articles.

When data were searched according to journals, a total of 200 journals contributed 1,441 articles, and the top 22 journals are shown in Table 2.

**Table 2:** Top 22 journals publishing papers related to transcranial direct current simulation in stroke The percentage was calculated based on the total number of 1,441. PLoS: Public Library of Science

Journal	Count (%)
Brain Stimulation	66 (4.58)
Frontiers in Human Neuroscience	66 (4.58)
Frontiers in Neurology	57 (3.96)
Stroke	47 (3.26)
Restorative Neurology and Neuroscience	45 (3.13)
Brain Sciences	40 (2.78)
Clinical Neurophysiology	33 (2.29)
Journal of Neuroengineering and Rehabilitation	33 (2.29)
Neurorehabilitation and Neural Repair	31 (2.15)
Frontiers in Neuroscience	30 (2.08)
International Journal of Stroke	22 (1.53)
Neuroscience Letters	22 (1.53)
Scientific Reports	22 (1.53)
Neuroimage	21 (1.53)
Archives of Physical Medicine and Rehabilitation	20 (1.39)
Neural Plasticity	20 (1.39)
PLoS One	20 (1.39)
Neurorehabilitation	19 (1.32)
Neuromodulation	18 (1.25)
European Journal of Neurology, Journal of Neuroscience, Trials (Each)	17 (1.18)

These 22 journals contributed 683 (47.43%) of the articles. The Brain Stimulation and Frontiers in Human Neuroscience each contribute 66 (4.58%) of the publications. Other prominent journals include Frontiers in Neurology, with 57 (3.96%), Stroke, with 47 (3.26%), and Restorative Neurology and Neuroscience, with 45 (3.13%) publications.

The language distribution of publications shows a predominant use of English, accounting for 1,572 (98.37%) of the studies, reflecting its role as the primary language of scientific communication. Other languages, such as German (16, or 1%), Spanish (three, or 0.19%), French (two, or 0.13%), and Polish (two, or 0.13%), contribute minimally, while Korean, Portuguese, and Russian each represent only one (0.06%) article.

There were a total of 200 affiliations among the authors, with a median of 12 (Q1-Q3: 8-19) publications per affiliation. As there might be multicentric studies, with each paper having multiple affiliations, the total number of publications from the institutions yields 3,354 publications. The top 20 affiliations are shown in Table 3.

**Table 3:** Top 20 affiliations of the authors publishing articles on transcranial direct current stimulation in stroke NIH: National Institutes of Health, USA: United States of America, IRCCS: Istituto di Ricovero e Cura a Carattere Scientifico

Affiliations	Count (%)
Harvard University	131 (3.91)
Harvard Medical School	119 (3.55)
Beth Israel Deaconess Medical Center	81 (2.42)
National Institutes of Health NIH USA	61 (1.82)
Johns Hopkins University	54 (1.61)
NIH National Institute of Neurological Disorders Stroke	54 (1.61)
University of London	54 (1.61)
Universidade De Sao Paulo	52 (1.55)
City University of New York Cuny System	51 (1.52)
University College London	50 (1.49)
Berlin Institute of Health	45 (1.34)
Charite Universitatsmedizin Berlin	45 (1.34)
Free University of Berlin	45 (1.34)
Humboldt University of Berlin	45 (1.34)
City College of New York Cuny	44 (1.31)
University of Gottingen	40 (1.19)
Spaulding Rehabilitation Hospital	38 (1.13)
Eberhard Karls University of Tubingen	35 (1.04)
Medical University of South Carolina	35 (1.04)
IRCCS Santa Lucia	33 (0.98)

**Figure 5:**
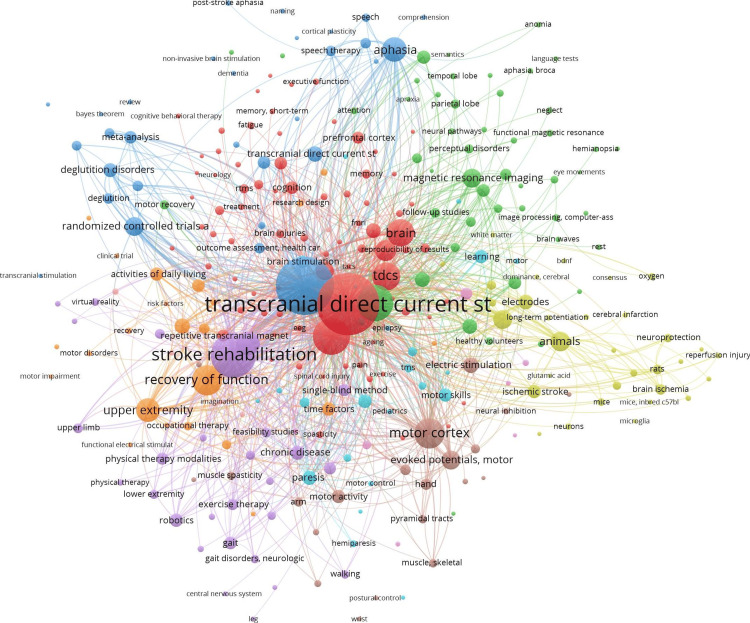
Co-occurrence of keywords network visualization of 1,300 PubMed-indexed article Source: The figure was generated in VOSviewer version 1.6.20

The co-occurrence network visualization, generated from PubMed literature, is shown in Figure 5.

The co-occurrence network highlights key research themes and relationships. The central nodes, such as “transcranial direct current stimulation,” “stroke,” and “stroke rehabilitation,” represent the primary focus on using tDCS for motor recovery and brain plasticity in stroke rehabilitation.

Discussion

In this study, we found that there was a growing scientific interest and broader adoption of tDCS in stroke research. The surge in publications indicates heightened research activity and the recognition of tDCS as a promising intervention in stroke rehabilitation. A previous study showed that there was a generalized upbeat from 2012 to 2021 [9]. This recognition underscores its promise as an innovative and effective therapeutic approach [10].

The subject-wise categorization of studies shows a clear emphasis on neuroscience, highlighting its foundational role in understanding the mechanisms of tDCS [11]. Clinical neurology and rehabilitation follow, underlining the clinical relevance of tDCS in treating stroke-related impairments and improving functional outcomes. This distribution points to the multidisciplinary nature of tDCS research, integrating basic science with clinical applications [12,13].

The global contributions from 69 countries reflect widespread interest in tDCS research, with the United States leading significantly, followed by Germany, China, and Italy. This indicates that high-income countries with robust research infrastructures are driving advancements in the field. A study by Sun et al. reported that high-income regions, such as North America and Western Europe, demonstrated extensive international collaborations. However, there was a lack of academic interaction among Asian nations and institutions [14]. Stroke prevalence and healthcare challenges vary significantly across regions, particularly in low- and middle-income countries, where the burden of stroke is often higher and resources are limited [15]. Geographically diverse studies can provide insights into the effectiveness of tDCS in varied healthcare settings, cultural contexts, and patient populations. Expanding research to underrepresented regions can also promote equitable access to innovative therapies, address regional disparities, and foster global collaboration for more inclusive and generalizable findings.

The dominance of a few major publishers, with Elsevier at the forefront, underscores the concentration of tDCS research dissemination within select publishing houses. Similarly, the contribution of a limited number of journals suggests the presence of specialized platforms that prioritize tDCS and stroke rehabilitation research. While this ensures visibility within focused audiences, expanding the scope to other journals could enhance reach and interdisciplinary collaboration. The overwhelming use of English as the primary language of publication reflects its role as the global medium of scientific communication [16]. The minimal contributions in other languages highlight the need for greater linguistic diversity to ensure accessibility for non-English-speaking researchers and practitioners.

The substantial number of author affiliations and publications per institution underscores the collaborative nature of tDCS research, often involving multicentric studies. This indicates a robust network of research institutions working collectively to advance the field. The co-occurrence network highlights the primary focus areas of tDCS research, such as motor recovery and brain plasticity in stroke rehabilitation. These central themes reflect the therapeutic potential of tDCS in addressing stroke-related impairments and its growing role in neurorehabilitation [17].

This study offers detailed insights into publication trends, subject categories, geographical contributions, and key research themes. By highlighting the dominance of neuroscience, clinical neurology, and rehabilitation, as well as the central focus on motor recovery and brain plasticity, the study underscores the multidisciplinary and evolving nature of tDCS research. However, there were some limitations of the study. A major limitation of the study methods is that it relied solely on two bibliographic databases, WOS and PubMed, which may have excluded relevant publications not indexed in these platforms [18]. However, we had access to only these two databases. The use of specific keywords in the search strategy might have overlooked studies that employ different terminology or less common methods for indexing tDCS research.

## Conclusions

The bibliometric analysis highlights a growing interest in tDCS for stroke rehabilitation, with a steady increase in publications. Research predominantly focuses on neuroscience, clinical neurology, and rehabilitation, emphasizing tDCS's role in stroke recovery and brain plasticity. The concentration of publications among major publishers and journals reflects the prominence of select platforms in advancing this field. However, the underrepresentation of research from developing countries calls for a more geographically diverse approach to ensure broader perspectives and equitable access to tDCS in different healthcare settings. Expanding research from low- and middle-income countries would contribute to a more balanced and inclusive global understanding of tDCS in stroke rehabilitation.
